# Effect of COVID-19 on antenatal care: experiences of medical professionals in the Netherlands

**DOI:** 10.1186/s12978-023-01587-y

**Published:** 2023-03-08

**Authors:** Carlotta Gamberini, Federica Angeli, Lucia Knight, Mariama Zaami, Salwan Al-Nasiry, Elena Ambrosino

**Affiliations:** 1 Institute for Public Health Genomics (IPHG), Department of Genetics and Cell Biology, Faculty of Health, Medicine & Life Sciences, Maastricht University, 6229 ER Maastricht, The Netherlands; 2 Research School GROW for Oncology and Reproduction, Maastricht University, 6229 ER Maastricht, The Netherlands; 3 School for Business and Society, University of York, York, YO105DD UK; 4 Division of Social and Behavioural Sciences, School of Public Health, Faculty of Health Sciences, University of Cape Town, Cape Town, 7700 South Africa; 5 School of Public Health, Community and Health Sciences, University of the Western Cape, Bellville, 7535 South Africa; 6 Department of Geography and Resource Development, University of Ghana, LG25 Accra, Ghana; 7 Department of Obstetrics and Gynecology, Maastricht University Medical Centre+, 6229 HX Maastricht, The Netherlands

**Keywords:** Antenatal care (ANC), COVID-19, Gynaecologists, Midwives

## Abstract

**Background:**

COVID-19 has greatly affected the delivery of all health care services globally. Antenatal care is one area of care that has been impacted, despite the fact that attending antenatal check-ups is essential for pregnant women and cannot be postponed. Little is known about how exactly ANC provision has changed in the Netherlands, or how the changes have impacted midwives and gynaecologists providing those services.

**Methods:**

This study used a qualitative research design to investigate changes in individual and national practice following the onset of the COVID-19 pandemic. The study involved a document analysis of protocols and guidelines for ANC provision to evaluate how those changed following the onset of the COVID-19 pandemic and semi-structured interviews with ANC care providers (i.e., gynaecologists and midwives).

**Results:**

Guidance was issued by multiple organizations, during the pandemic, on how to approach the risk of infection in pregnant women, recommending several changes to ANC to protect both pregnant women and ANC providers. Both midwives and gynaecologists reported changes in their practice. With less face-to-face consultations happening, digital technologies became critical in the care of pregnant women. Shorter and fewer visits were reported, with midwifery practices adjusting their guidelines further than hospitals. Challenges, with high workloads and lack of personal protective equipment were discussed.

**Conclusions:**

The COVID-19 pandemic has had an immense impact on the health care system. This impact has had both negative and positive effects on the provision of ANC in the Netherlands. It is important to learn from the current COVID-19 pandemic and adapt ANC, as well as health care systems as a whole, to be better prepared for future health crises and ensure continuous provision of good quality care.

**Supplementary Information:**

The online version contains supplementary material available at 10.1186/s12978-023-01587-y.

## Plain Language summary

COVID-19 has affected the delivery of healthcare services globally. Antenatal care is one of the healthcare services that has been impacted on a global scale. Little is known about how antenatal care provision has changed in the Netherlands during the pandemic period. Our project focuses on examining the effects of COVID-19 on existing antenatal care protocols, as well as the impacts on antenatal care providers, such as midwives and gynaecologists. This knowledge can be beneficial in adapting antenatal care provision in times of health emergencies, to be better prepared and more resilient. This research uses a qualitative approach to investigate changes in practice following COVID-19 pandemic. It involves 20 antenatal care providers, working in the Netherlands, which took part in semi-structured interviews, and 9 national protocols and guidelines which were analysed. This study indicates that antenatal care changed at different levels in the Netherlands. Many changes show that antenatal care is an essential service, which should not be cut back, but it should be implemented, to be prepared for a future health emergency.

**Supplementary Information:**

The online version contains supplementary material available at 10.1186/s12978-023-01587-y.

## Background

Since the first case of the Coronavirus-Diseases 2019 (COVID-19), an infectious disease caused by the SARS-CoV-2 virus, was identified and reported in December 2019, the infection has spread rapidly across the globe. The disease, classified as a pandemic by the World Health Organization (WHO), predominately affects the respiratory system and can lead to a variety of clinical presentations, ranging from an asymptomatic state to severe [1].

Certain individuals, such as older people, people suffering from cardiovascular diseases, diabetes and other co-morbidities, are at a higher risk of adverse outcomes if infected [2]. Physiological and immunologic changes during pregnancy may also result in a higher susceptibility to viral infections, particularly respiratory viruses, leading to maternal and foetal morbidity and mortality [3]. To date, most pregnant females infected with COVID-19 have developed milder symptoms including fever, cough and dyspnoea and mild pneumonia [4–9].

COVID-19’s impact on other neonatal and obstetric outcomes remains unclear. According to an international initiative on COVID-19 and maternal and child health, the preterm birth rate among pregnant women infected with COVID-19 is approximately 17% higher than among uninfected pregnant women [6], with initial concerns that COVID-19 could be linked with foetal growth restriction [10]. To date there is little evidence to corroborate these claims, and Huntley et al. [11] found no significant difference in the number of small for gestational age infants born from COVID-19-positive versus COVID-19-negative mothers. Further, Mullins et al. [12] found the number of stillbirths and early neonatal deaths to be comparable to historical data, when they reviewed United States (US) and United Kingdom (UK) registry data on 4005 pregnant women with COVID-19.

COVID-19 has also been reported to have indirect negative impact on maternal and new-born health services. As a result of this pandemic, health services around the world have been subject to an unprecedented level of stress and strain, leading to the restructuring of care delivery [13]. This is exemplified in the work by Roberton and colleagues [14], that estimated a reduction in antenatal care (ANC) delivery by at least 18% in 118 low- middle-income countries (LMICs). Similarly, a Ugandan study [15] has shown that the number of ANC attendances decreased during the three-month lockdown. Moreover, according to an International Planned Parenthood Federation survey, many clinics and community-based care outlets that provide ANC services had to close across 64 countries, and the facilities that remained open were overwhelmed. This was particularly severe for those in LMICs [16]. In India, a 45.1% reduction in hospital-assisted deliveries was observed along with that of 32.5% of pregnant women who received adequate ANC [17]. ANC is important in the prevention, detection and treatment of health problems during pregnancy and in ensuring the good health of mother and baby. Regular ANC is essential in any healthcare system. Past and current epidemics have been directly and indirectly affecting ANC delivery with associated adverse pregnancy outcomes [18].

However, pandemics do not only affect the health care systems of LMICs but also those of high-income countries (HICs). In the UK research highlighted a shortage of midwives due to their reassignment to COVID-19 care, being sick or required self-isolate [19]. Since the start of the pandemic, the UK, along with other countries, such as France, have suspended most face-to-face consultations replacing them with telephone and online consultations [20]. Evidence from the UK suggests that a 79.1% reduction of face-to-face antenatal consultations [21] may have affected the mother's mental health and well-being [22]. These findings also suggest that reduced personal and in person support from ANC providers could reduce interventions for ANC complications [20].

Overall, the full implications of changes in care for babies and mothers have remain unclear, although increasing evidence is becoming available. Results of a study examining the impact of telehealth (sharing of health-related services via digital and telecommunication technologies) on maternity care in Victoria (Australia) found that, compared to conventional ANC, integrated telehealth ANC had no significant impact on pregnancy outcomes, including foetal growth restriction and preterm birth.

While there is a growing amount of literature on the impact of COVID-19 on ANC in some countries, such as the UK, little has been published on how the pandemic has impacted ANC in others, such as the Netherlands. Unlike in neighbouring countries, in the Netherlands, ANC is provided at all care levels [23]. The most ANC is provided at primary care level by midwives or general practitioners (GP) to pregnant women with the lowest risk of complications. Midwives are independent professionals and support women during pregnancy, childbirth and post-partum. For women with a risk of complications, midwives refer women to the hospital where gynaecologists and specialized midwives provide secondary care, with academic hospitals also dealing with complex clinical cases [23].

In the Netherlands, COVID-19-related guidance to care provision has been released by the Dutch Society for Obstetrics and Gynaecology (Nederlanse Vereniging voor Obstetrie en Gynaecologie, NVOG) and other professional organizations. Little is known about the extent to which these guidelines have been implemented in real life and have impacted the provision of care. A recent digital survey published by van Manen et al. [24] provided insights into midwives’ perceptions on ANC changes in the Netherlands during the pandemic. The survey found that approximately 50% of maternal health care providers felt policy changes led to a compromise in the safety of healthcare provision, 50% also felt that it was beneficial to have fewer face-to-face consultations [24]. Although this study provides helpful insights into the Dutch situation, the survey only captured limited information and excluded gynaecologists.

The current study investigated how the COVID-19 pandemic affected provision of ANC in the Netherlands, focusing on experiences of midwives and gynaecologists and ANC providers over the course of two years, and different waves (the surge in new cases happening during the pandemic). The main objectives of this study were: to understand how guidelines around ANC provision have changed in response to the COVID-19 pandemic in the Netherlands; and to understand how provision of ANC care by midwives and gynaecologist has been adapted following the COVID-19 pandemic in the Netherlands. In order to support healthcare system resilience and an effective adaptation of ANC provision in times of health emergencies, it is important to examine how current guidelines and recommendations have impacted provision of care at the local level.

## Methods

### Study design

This study used a qualitative research design involving two sources of data: (i) a document analysis of protocols and guidelines for ANC provision to evaluate how they changed following the onset of the COVID-19 pandemic; (ii) semi-structured interviews with ANC care providers (i.e., gynaecologists and midwives) to investigate changes in individual practice following the onset of the COVID-19 pandemic.

This study was part of the larger international project “Strengthening ANC Resilience in the Face of Pandemics (SARA)” conducted by Maastricht University (the Netherlands), the University of York (the UK), the University of Cape Town (South Africa) and the University of Ghana (Ghana).

This study aligns with the Consolidated criteria for reporting qualitative research (COREQ) [25].

### Participants

**Fig. 1:**
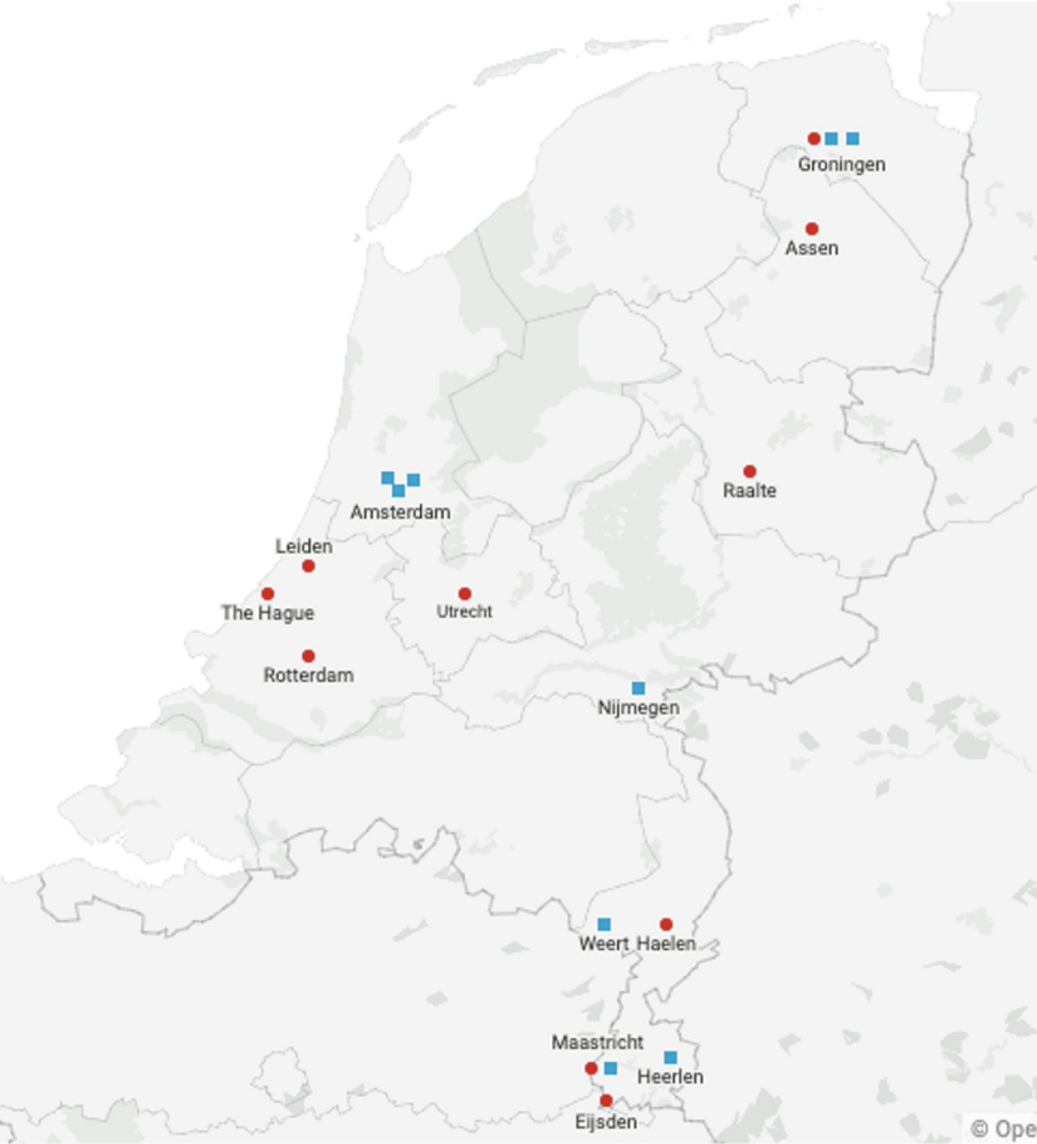
Distribution map of interviewees. Each red circle represents a midwife, each blue square a gynaecologists

Participants were recruited from 20 midwifery practices and hospitals across the Netherlands (Fig. 1), and 11 midwives, and 9 gynaecologists in active employment and working in community practices or hospitals within the Netherlands were interviewed. They were invited to participate by: (i) e-mailing individual practices or providers; (ii) advertising the research project via relevant organizations. The main criterion for recruitment was willingness to participate, ability to speak English and having ANC work experience during the pandemic period. No incentives were provided to participants.

### Data collection

#### Document search

Documents were identified using the query search (antenatal care OR ANC) AND (Netherlands OR NL) AND (COVID-19 OR COVID-19 pandemic), and keywords such as “antenatal care”, “Netherlands” and “COVID-19” were used to look for a comprehensive overview of guidelines, using relevant national or international organizations’ websites, as well as PubMed and Google Scholar. The guidelines selected had to meet certain inclusion criteria: (i) contain information about ANC services in the Netherlands, (ii) be published from 15 years previous to the start of the COVID-19 pandemic (from 2004) onwards and (iii) be evidence-based or peer-reviewed. Documents from the Royal Dutch Organization of Midwives (Koninklijke Nederlandse Organisatie van Verloskundigen, KNOV), the Dutch Organization for Gynaecologists and Obstetricians (Nederlandse Vereniging voor Obstetrie en Gynaecologie, NVOG), the National Institute for Public Health and Environment (Rijksinstituut voor Volksgezondheiden Milieu, RIVM), and the Dutch College of General Practicioners (Nederlands Huisarten Genootschap. NHG) were examined. Documents, in either English or Dutch, were collected between May 2021 and May 2022. Guidelines that were in Dutch were translated into English using a web-based translation tool (Google Translate), and subsequently verified by a Dutch-speaking peer.

#### Semi-structured interviews

The interviews followed a semi-structured approach with a predefined interview guide (Additional file 2) developed with and reviewed by experts in ANC and qualitative research, to assure relevance and help eliminate leading questions. The interview guide was designed with study aim and objectives in mind.

Interviews were conducted in English, online, at the most convenient time for the interviewee and the audio component was recorded using a digital recorder, and lasted approximately 30 min. Study participants were interviewed by one of the research team member and author (CG) with the support of graduate students.

### Data analysis

#### Document analysis

The documents were analysed using content analysis, involving a first step of skimming through the documents for a superficial examination, followed by full reading for a thorough examination. Finally, with the use of a data collection and interpretation tool (Additional file 1) content and thematic analysis was performed, information was organized into categories followed by pattern recognition with emerging themes.

#### Semi-structured interviews

Following transcription, interviews were read multiple times while recording notes about possible codes. The three-step methodology by Gioia et al. [26] was used to conduct the thematic analysis of interview transcripts. In the first step, transcripts were coded using both inductive and deductive codes. In order to do so, the software Atlas.ti was utilized for creating codes and identifying common themes. In the second step, emerging themes suggested concepts that might help describing and explaining the observed phenomena. To refine the specifics of each theme, and the overall story the analysis tells, clear definitions and names for each theme were generated. In the last step, similarities across second-order themes were ascertained, which allowed for subsequent grouping of second-order themes into aggregate dimensions. A thematic ‘map’ of the analysis was generated.

### Ethical approval and informed consent

Ethical approval for this project was granted by the Maastricht University Medical Center Medical Ethics Committee (METC, Medisch Ethische Toetsings Commissie) under registration number METC 2021-2517.

Prior to the interviews, participants were asked to read the participant information sheet (Additional file 3) and to sign the informed consent form (Additional file 4) electronically. After consent was provided, recording could start. Data from the interviews was transcribed, pseudonymised and stored to fulfil local data protection regulation.

## Results

### Document analysis

The guidelines (n = 9) examined for the document analysis were published by professional organisations based in the Netherlands. The guidelines selected were from the following professional organisations: (i) NVOG (n = 3); (ii) KNOV (n = 4); (iii) RIVM (n = 1); and (iv) NHG (n = 1).

### Protocol pre COVID-19 pandemic

Prior to COVID-19, midwifery practices in the Netherlands had some flexibility on how to set up ANC [27]. The KNOV recommended all practices to follow an antenatal schedule, comprising between 10 and 16 consultations, and one or two group information meetings [28] (Table 1).

**Table 1:** ANC consultation schedule pre COVID-19 revised from [27–31]

Trimester	Gestational Age	Examinations & Activities
First trimester	6–8 weeks	Introduction, information about ANC practice, screening for risk factor
	8–10 weeks	Prenatal screening: ultrasound, medical history, weight, BMI, RR, blood test, OGTT
	10–13 weeks	Second consultation when necessary
Second trimester	14–17 weeks	(Possible) Group Information about the pregnancy period
	14–26 weeks	2–3 consultations depending on need: SEO and GUO, RR, foetus cardiac activity, growth and foetus movements. Provide information on preeclampsia complaints and imminent preterm birth
Third trimester	27–40 weeks	6–8 consultations depending on need: RR, foetus cardiac activity and growth, foetus movements and engagement, haemoglobin levels. Discuss birth plan, labour and delivery
	41–42 weeks	1–3 consultations depending on need: RR, foetus cardiac activity and growth, foetus movements and engagement. Discuss possibility of labour initiation
	30–35 weeks	(Possible) Group Information about the labour and post pregnancy period

*BMI* Body mass index, *RR* Respiratory rate, *OGTT* Oral glucose tolerance test, *SEO* Structureel echoscopisch onderzoek, Structural ultrasound examination, *GUO* Geavanceerd ultrageluid onderzoek, Advance ultrasound examination

For those attending secondary and tertiary care, the first contact in the hospital was usually the intake at 10–11 GA weeks. That appointment was accompanied by an ultrasound and counselling about the option of performing the Non-Invasive Prenatal Test (NIPT, a blood test to investigate foetal chromosomal abnormalities, such as Down syndrome, Edwards and Patau syndrome) and a blood test to investigate: ABO blood group, Rhesus antigen, blood group antibodies, haemoglobin level, thyroid function, glucose level, and diagnose syphilis, Hepatitis B and HIV infections [29, 30].

Prior to the COVID-19 pandemic, the NHG and KNOV also issued joint guidance about infection, prevention and control. Both organizations instructed practitioners to wear short-sleeved clothing, and personal protective equipment (PPE) in high-risk situations, for example with HIV patients [27, 31]. Sterile gloves were recommended during any invasive procedure. Guidance on mask use was at midwives’, gynaecologists’ and general practitioners’ discretion: the NHG [31] suggested providers wear a respiratory protection mask (type FFP2) if there was a risk of transmission of microorganisms that could lead to a serious infectious disease.

### Protocols after the start of the COVID-19 pandemic

As a consequence of the COVID-19 pandemic, guidance was issued by multiple organizations on how to approach the risk of infection (Table 2). The KNOV recommended several changes to ANC to protect both pregnant women and midwives [32]. Firstly, midwives were recommended to call clients prior to appointments to explain hygiene measures, to triage for COVID-19 symptoms, and to advise pregnant women to come to consultations alone. The KNOV also advised midwifery practices to change their ANC schedule, recommending a reduction in the number of face-to-face consultations to seven. Midwives were encouraged to switch to telephone/video consultations where possible, and to only offer medically necessary ultrasounds [32].

**Table 2:** COVID-19 changes in schedule revised from [32–35]

	KNOV	NVOG	RIVM
Schedule	Recommending a reduction in the number of face-to-face consultations to seven Stopping regular visits postnatal when possible	No major changes to ANC schedule. Recommending monitoring foetal growth in the third trimester with two ultrasounds from 28-weeks	–
Telemedicine	Encouraging to switch to telephone/video consultations where possible, and to only offer medically necessary ultrasounds	Recommending the use of telephone or video counselling when discussing the form of delivery	Recommending use of digital technologies in the form of counselling via telephone (counselling conversation for screening for Down syndrome, Edwards syndrome and Patau syndrome could take place via phone)
Physical distancing	Only one person can be present at a home delivery, in addition to the maternity nurse and obstetrician	Limited the number of people is allowed during an appointment. The women should come alone, an exception was made for the 10-week and 20-week scan, at which partners were allowed	–
Infection control (1st) ANC provision	Call clients prior to appointments to explain hygiene measures, to triage for COVID-19 symptoms, and to advise pregnant women to come to consultations alone	Women with mild COVID-19 are allowed to deliver their baby at home, but additional monitoring of respiratory rate is recommended	Physical distancing, hand washing, and working from home. Doctors and patients are required to keep 1.5 m distance, if possible, to not shake hands, apply hand hygiene and use paper towels. The practitioners are asked to use a surgical mouth-nose mask but make their own decision whether the patient has to use such a mask

Similarly, the NVOG limited the number of people allowed during an appointment. In their guideline, the women should come alone, with an exception made for the 10 GA week and 20 GA week scan, at which partners were allowed [33]. Furthermore, the guideline by the NVOG also recommended the use of telephone or video counselling when discussing the form of delivery, in addition to postpartum follow up appointments, among others [33]. Concerning delivery, the KNOV advised that only one person be present at a home delivery, in addition to the midwife. Postnatally, the KNOV also suggested stopping regular visits, and replacing them only with visits according to medical needs [32].

The NVOG [34] suggested no major changes to ANC for women with COVID-19 during pregnancy. However, they did recommend monitoring foetal growth in the third trimester with two ultrasounds from 28 GA weeks on in these patients [34]. In those with severe infection, ANC should be transferred to a hospital setting, whereas for mild infection, care could remain in the community, also depending on other co-morbidities. Furthermore, women with mild COVID-19 were allowed to deliver at home, but additional monitoring of respiratory rate was recommended, using a Modified Early Obstetric Warning Score (MEOWS, designed to allow early recognition of physical deterioration in pregnant women by monitoring their physiological parameters) to help guide escalation decisions: if the MEOWS score was ≥ 3, midwives were recommended to refer their patient to the hospital [34]. Overall, the approach used by the KNOV is similar to that used by the NVOG.

The RIVM [35], which was mostly concerned about general pandemic guidelines not specific to ANC, recommended pregnant women follow standard measures including physical distancing, hand washing, and working from home. The guideline published by the RIVM stated that doctors and patients were required to keep 1.5 m distance, if possible, to not shake hands, apply hand hygiene and use paper towels. In addition to that, the practitioner was asked to use a surgical mouth-nose mask but make their own decision whether the pregnant women had to use such a mask.

In most guidelines, the use of digital technologies was mentioned in the form of counselling via telephone. For example, in the recommendation by the RIVM regarding screening for Down syndrome, Edwards’s syndrome and Patau’s syndrome, it was written that counselling conversations could take place via phone.

### Interviews with practitioners

As part of this study, eleven midwives and nine gynaecologists were interviewed (Table 3). All midwives worked in shared community practices spread across the Netherlands. Of them, 4 worked in urban areas, while the remainder worked in semi-urban settings (Fig. 1). They all had between 1.5 and 35 years of experience in ANC.

**Table 3:** Characteristics of interviewees

Interviewee	Profession	Location	Type of contract
G1	Medical Doctor (not in specialist training, ANIOS)	Zuyderland hospital, Heerlen	Full time
G2	Gynaecologist	Amsterdam UMC	0.9
G3	Gynaecologist	St. Jans Gasthuis, Weert	Full time
G4	Obstetrician	University Hospital Nijmegen	Full time
G5	Gynaecologist	Amsterdam UMC	0.8
G6	Professor in Obstetrics	Amsterdam UMC	0.9
G7	Perinatologist	University Medical Center Groningen	Full time
G8	Perinatologist	University Medical Center Groningen	Full time
G9	Gynaecologist	University Medical Center Groningen	Full time
M1	Midwife	South Holland	Full time
M2	Midwife	Limburg	Full time
M3	Midwife	Drenthe	Less than full time
M4	Midwife	Limburg	Full time
M5	Midwife	South Holland	Full time
M6	Midwife	Utrecht	Full time
M7	Clinical Midwife	Groningen	0.9
M8	Midwife and midwifery teacher	Groningen	Part time
M9	Midwife	Raalte	Full time
M10	Midwife	Maastricht	Full time
M11	Midwife	Rotterdam	Full time

The gynaecologists interviewed worked at four different Dutch hospitals. The majority of them at academic hospitals (n = 7; Amsterdam University Medical Centre University Hospital Nijmegen, University Medical Center Groningen, Maastricht University Medical Center), two at peripheral hospitals (n = 2; Zuyderland Hospital, Heerlen and St. Jans Gasthuis, Weert). Their working experience spanned from 4 to 16 years. Table 3 details the characteristics of interviewees.

**Fig. 2:**
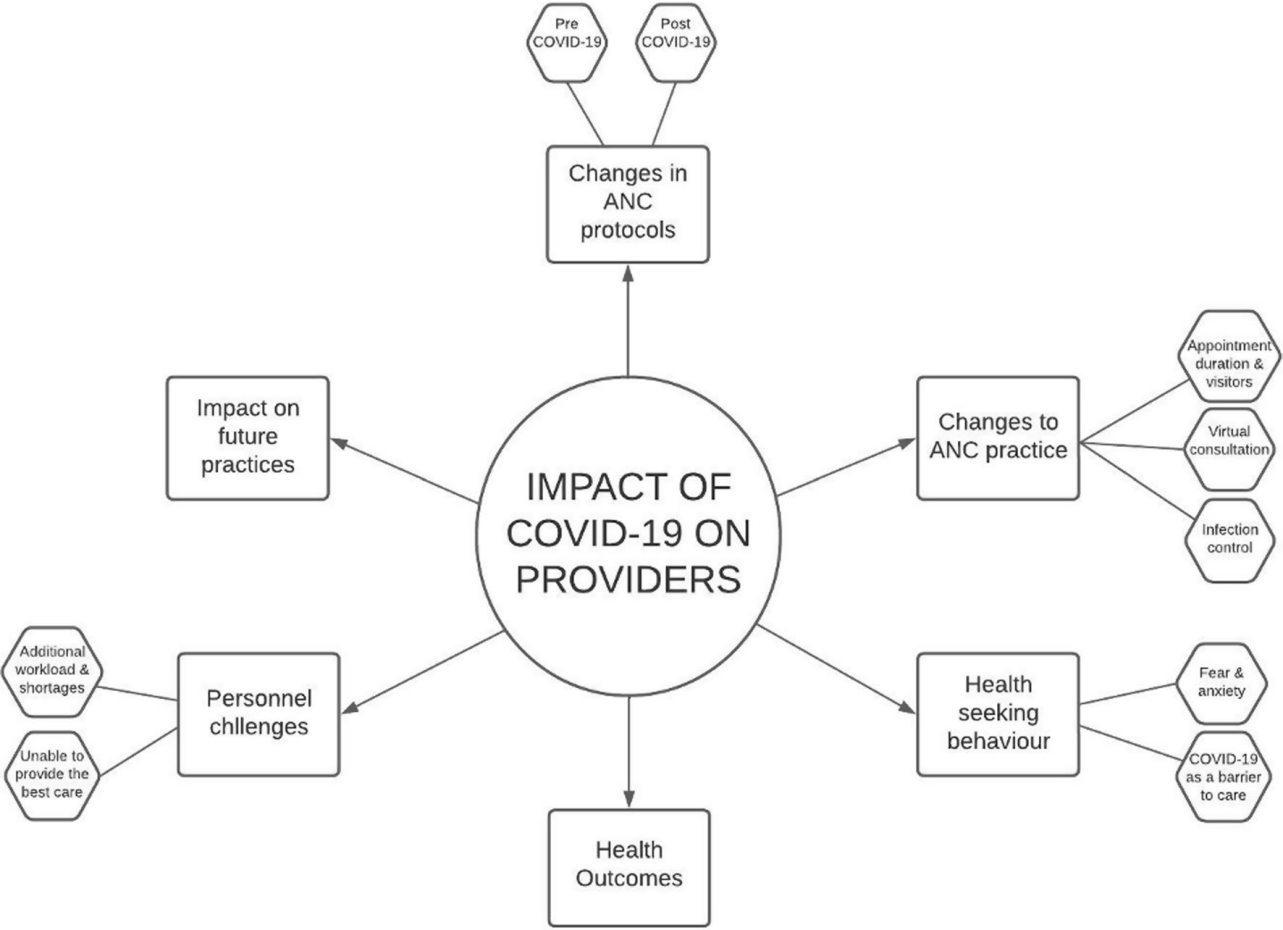
Themes and subthemes from providers’ interviews

Analysis of the interview transcripts revealed five main themes and additional subthemes, as shown in Fig. 2.

#### Changes in ANC protocols: pre and post pandemic

##### Schedule pre COVID-19

Midwives were asked during the interview process about the local ANC schedule used in their practices prior to the COVID-19 pandemic. There seemed to be variability in consultation numbers (M4-M6) between practices, but all seemed to follow KNOV guidance. Again, midwives followed KNOV guidance concerning ultrasounds, although there was variation in total ultrasound numbers (M2-M4-M9) between midwives.

Primary care midwives would usually be responsible for low-risk pregnancies, with higher-risk pregnancies transferred to secondary or tertiary care, for example:

“If the mother has complications like hypertension or diabetes or anything in the general health, then she will be referred to the hospital and then the ANC will be in the hospital as well” (M7).

Similarly, gynaecologists have described the normal schedule that follows the national guidance, from KNOV. All participants have highlighted that this schedule is highly dependent on the health and clinical status of the woman (G1–G9).

“Yes, the midwives always do the first check-up in our region. Also, […] if already known that it will be hospital case because of a pre-existing conditions or risks. But that is an agreement we have made with all the midwife practices in our region” (G4).

##### Schedule post COVID-19

During the COVID-19 pandemic, midwives reported multiple changes to ANC protocols. They said they received guidance at a national level, from the government and their professional organization, the KNOV. However, there were some adjustments at a local level to national protocols, depending on the COVID-19 situation in that area (M3, M6, M7, M8, M9).

The number of in person check-ups were reduced, sometimes replacing them with videophone or telephone check-ups.

“The KNOV had…some changes, and we removed two consults in early pregnancy. So, before 23 weeks, we saw women two times less, and we had shorter consults” (M6)

During the COVID-19 pandemic, gynaecologists reported changes to ANC protocols, which in certain instances had to be introduced in a very short amount of time (G4). ANC was prioritized, due to the urgent and not delayable nature of the care provided.

Comparably to midwives, gynaecologists received guidance at a national level, from both the government, and from their professional organization, the NVOG (G1, G9). All hospitals debated the necessity of the visits as planned in the normal schedule. In some cases, it was decided that high-risk patients were to come only every four weeks and low-risk patients even less (G1). One participant highlighted that they kept most of the schedule as normal as possible, but cancelled the 13-week appointment (G2). One interviewee mentioned that, while they tried to keep women at home as much as possible, they kept a similar schedule for high-risk women (G7, G8). Some hospitals skipped the 16-week and 24-week appointments (G5, G6). Most interviewees addressed the difficulties to organize and schedule care around the new protocols and referred to the delays that the pandemic brought (G8). Moreover, the focus on home monitoring for low-risk patients was mentioned. However, the importance of face-to-face care was highlighted. One participant mentioned the hospital-based guideline of decreasing care by 20–30% (G1) per department.

#### Changes to ANC practice during COVID-19

Following the adaptation in care protocols, many processes changed during the pandemic period, including the use of digital technologies, hygiene measures and more in general the length and number of appointments.

##### Appointment duration and visitors

Prior to the pandemic, most ANC consultations were approximately 15 min.

However, during the pandemic, face-to-face ANC appointments were either replaced with video or telephone calls, or the appointment duration shortened. The reduction in the length of face-to-face appointments was triggered by a change in consultation structure in some practices.

“Everything was shorter. It was horrible. I think they were in for five minutes, four, and then we call them” (M6)

In one practice, midwives tried to keep face-to-face appointment times at 15-min, but because extra time was needed for cleaning, the time available for dealing with clinical problems was de facto reduced (M3).

The pandemic also led to changes in the number of visitors allowed to attend ANC appointments. Many clients had to attend consultations alone, without their partners.

“We had to change a lot because normally all the kids come in, so we have a lot of toys for the kids. And we have, like coffee and tea, and snacks for everybody. So, in the waiting room, we have to get rid of everything, so that nobody was alone or allowed to touch anything” (M2).

According to several midwives, the biggest challenge for pregnant women was having to attend ANC appointments alone, or with fewer family members and friends, than prior to the pandemic. Many practices received complaints about this (M4).

Conversely, gynaecologists did not experience a shortening of the appointment duration, during the pandemic, some appointments were replaced with video or telephone calls (G1, G2, G7, G9). It was highlighted that with more consultations happening via phone, the quality of care was compromised due to the inability to see facial expression (G1, G3). Moreover, sometimes the phone numbers were missing, or the phone wasn’t answered. Then the care provider had to stay longer and try calling again at the end of the workday. Other participants mentioned that they did not use the video and phone consultations for the pregnant population because face-to-face visits were prioritized. The number of people that were allowed for each appointment was limited as women were encouraged to go alone to each visit, with partners sometimes allowed to the 20-week scan (G1), comparable with midwives’ experiences.

##### Virtual consultation

In some cases, face-to-face appointments were entirely replaced with virtual consultations, due to COVID-19 restrictions. Many midwives started using video and telephone calls to communicate with patients, rather than seeing patients face-to-face (M5, M4).

“We try to do some check-ups by phone. But it's hard because you want to feel the belly and check the growth of course. So, it's not always appropriate to do it by phone only. And so, it mainly results in the postpartum check-ups. We did them by phone most the times, because it's only a conversation and not really a help not really a physical check most of the times so that is something we still do a lot of those check-ups by phone.” (M7).

For some practices, there were initial logistical problems in setting-up video calling services and they had to rely on telephone calls initially. For example, some practices did not have cameras installed, or have encrypted internet connections to ensure confidentiality of patient information.

There were disadvantages to using video calling for this purpose. For instance, it was more challenging to identify complications and health status of the mother and child, such as increased blood pressure in the mother or new-born jaundice (M1).

On the other hand, other hospitals implemented the digital technologies for every appointment that was not strictly necessary to do in person in gynaecologists’ practice. Most video and phone consultations were done for postnatal check-ups, seldom for ANC (G1, G2, G7). Partly because the perception of the practitioners was that the patient felt insecure with the digital technologies and because most appointments during ANC have a physical component that could not be done remotely or via home monitoring (G6).

“And I think that also by phoning them they do not always feel that they can ask you all of the questions, because when you are just facing each other, when you are sitting in a room you can see that there is time enough and that I’m relaxed and that I want to answer all of the questions. But maybe, if they only hear my voice they feel like: Oh, she’s stressed. Oh, she’s busy. I don’t what to keep her up. Yeah, I will just say that I have understood everything and let’s move on.” (G1).

##### Infection, prevention and control and PPE

The pandemic led to wider use of PPE in community midwifery practices. However, different practices introduced PPE at different times depending on availability, with many respondents reporting challenges.

In fact, many midwives had only limited equipment and supplies, which were inadequate to meet midwife requirements. Masks were initially difficult to procure, and some midwives adapted to the situation by using substitute PPE (M5, M6).

“With regular patients, we would wear gloves and the surgical mask. I think that was only in September [2020] that they started to wear that standardly.” (M1)

Respondents reported feeling unsupported by the government in terms of PPE provision at the start of the pandemic, as private workers, community midwives did not receive the same help as midwives working in hospitals, and there was seemingly a lack of national co-ordination to ensure community midwives received PPE (M4-M6).

Most respondents seemed to procure PPE from different non-Governmental sources, sometimes at considerable expense.

“There were no masks for midwives. We have some contacts. And we managed to arrange in the beginning, 20,000 masks … with China. It costs us 23,000 euros” (M6)

Most midwifery practices had to adopt their cleaning regimes in response to COVID-19.

Midwives also had to adjust their practices to minimize the risk of COVID-19 transmission. For example, they had to try to improve ventilation within their surgeries, make changes to the waiting room, the remove of reading materials, and toys from waiting rooms (M4, M8).

In a similar way, also gynaecologists were required to expand and strengthen hygiene measures, for example keeping distance from each other, keep spaces cleaned and sanitized and wearing facemasks and other PPE (G1–G9).

Although facemasks were implemented only in the second wave of the pandemic, they had a considerable impact on the relationship between caregiver and patient. Facial expressions were much more difficult to read and understand. In addition to the physical distancing, facemasks made it even more difficult for the caregiver to form an emotional connection with the patient. This led to caregivers feeling generally less connected to their patients (G1, G3, G5).

#### Health seeking behaviour

##### Fear, anxiety and mental health

At the start of the pandemic, some midwives reported clients were more anxious and called more frequently for advice and support. This was often in response to changes in national guidance, or media coverage about pregnancy during COVID-19 (M1, M2, M5).

Midwives reported clients had less social support, were lonely and often isolated, which may also have affected health-seeking behaviour (M8).

“A lot of people feel, felt alone. So that's why also our consultations took really a long time, because they wanted to talk about how they are feeling.” (M2)

Gynaecologists reported patients changing their behaviour when faced with challenges related to the COVID-19 pandemic. It was mentioned that the patients felt anxious and fearful because of the lack of information about the virus early in the pandemic (G1). Women felt anxious and scared to go to the hospital and be infected by SARS-CoV2, and as a result they preferred staying home instead of going to their visits, when not necessary. The fear would also increase in the case of women with history of other diseases. In addition, fewer women called or came to the hospital with minor complaints (G8, G9).

Gynaecologists also reported that because of the pandemic, women had less social support, they were lonely and sometimes isolated, worsening their mental health. Furthermore, some patients felt insecure due to limited physical contact with the caregiver.

“I think they suffered in the sense that being pregnant is now a solitary issue, not a couple’s issue. (…) In the beginning, mainly, women didn’t want to get out of the house, so they postponed their controls” (G7).

Some participants observed a difference in reactions depending on patients’ cultural and educational background. Interviewees mentioned that people with a medical background and general higher educational background were more understanding of rules. On the other hand, patients, and patients’ partners from a lower social and educational background had more issues with following the rules. Additionally, people from a different cultural background had difficulties with the guidelines.

“Our foreign, (…) and they do not understand, and they just show up every time again, with a partner, but also because of the need for translation or something. So that, that group was quite difficult to address concerning those existing rules or accompanying rules, but for the rest it was quite ok.” (G3).

##### COVID-19 as a barrier to care

As the pandemic progressed, client behaviour changed and some midwives expressed concerns clients were not accessing their services, with COVID-19 acting as a potential barrier to care. Respondents reported clients were fearful of getting COVID-19 from midwives and therefore avoided contacting them with red flag symptoms (M9).

“I cannot say for sure, but the patients were indeed stressed and afraid for their baby. They thought something would happen to their baby if they were infected. Because of this fear and stress, they even suggest doing video calls instead” (M11).

A respondent gave an example of one couple who persistently refused to have face-to-face contact with a midwife, and eventually were found to have gestational diabetes (M1).

Some clients viewed the changes to ANC services negatively, as they felt they were not receiving the same level of quality care as prior to the pandemic (M1, M6, M9).

However, most respondent have also reported the women to be understanding of the changes implemented to ANC services (M3).

In a similar way, according to the gynaecologists, the health seeking behaviour of pregnant women changed. It was highlighted that the changes were very dependent on the individual patient, but that overall less women came early in the pandemic. Women also preferred the video calls. On the other hand, some interviewees mentioned that the health seeking behaviour of the patients did not change during the pandemic (G1, G3, G9).

Most of the pregnant women understood the change in rules and guidelines at first, but the longer the pandemic went on, the smaller the understanding of the rules was. Sometimes people got angry, which led to discussions with the practitioners. These situations were sometimes not easy to handle for the caregiver (G5).

##### Health outcomes

Based on observations, most midwives and gynaecologists did not observe many changes in the health outcomes of pregnant women or their babies. However, many providers were hesitant to discuss this topic in the absence of concrete audit data (M1, M3, G11).

Some midwives thought there were less premature babies, but overall, it was quite hard for them to judge, as they do not usually see high-risk pregnancies (M3, M8, M10).

### Challenges

#### Workload and PPE

Some midwives reported additional workload during the pandemic. For example, an interviewee had to do additional work, while off-duty, to maintain up to date with current guidelines.

“In my free day or evening, then we get this new information. And then I have to read that, when I was not on duty. I didn't like that, because I thought okay, that’s my day off.” (M5).

Another also found the PPE to be burdensome:

“The big change is that we had to protect her [and] ourselves…do a birth in one room for 12 h with you mask …it's not the best thing.” (M5)

Moreover, a respondent reported feeling unsupported by the government in terms of vaccination availability.

“The hospitals were vaccinated, and we try, we tried, but it was a shortage of vaccines… it was not coordinated, at national [level].” (M6)

Gynaecologists faced additional challenges with the shift in schedule. Special care needed to be provided for COVID-19-positive and suspected COVID-19-positive patients. These required special rooms, extra personal protection equipment and more careful handling in general.

“We had to consider everybody who had even the slightest complaint as suspicious. So, we had a special room for that, where we had a personal protective equipment outside the door. And we had to get dressed in the hall, go in and then undress before going out. And wipe all the surfaces down.” (G4).

Additionally, medical personnel were shifted to COVID-19 care. This shift created a shortage that at times posed a challenge for maintaining standard care, including for the pregnant population. With the shortage of people also came a shortage of medical equipment. Especially early in the pandemic, participants reported shortage of protective equipment. However, because of the prioritization of ANC the thread never caused any real problems (G2, G4).

“We had to work at the COVID ward as well to help out there, so our working schedule was quite restricted so that’s why we only met patients who really had to come and out schedule was altered” (G3).

Some participants mentioned the additional challenge of surgical guidelines that resulted in non-urgent surgeries being placed on a waiting list and the requirement of a negative COVID-19 test, when going to the operating room (G2, G6). This presented a challenge for the pregnant population, who had to go to the operating room, because they would be treated as if they were COVID-19 positive if they did not have a negative test result. These rules led to delay in care in some cases (G6). The participants criticized these guidelines because the availability of COVID-19 tests was limited at the time, and they felt that guidelines protected the medical personnel more than the pregnant patient and could have led to negative pregnancy outcomes (G2).

##### Unable to provide the best care

During the pandemic, there was an impact on job satisfaction. The reasons were multifactorial and included the use of PPE, a lack of face-to-face contact with clients, and the impossibility to provide optimal care (M11, M3).

Most gynaecologists have also touched upon the dissatisfaction of their work, and their inability to provide the best and optimal care to their patients, due to all the factors described above, lack of face-to-face contact, shortage of personnel and PPE (G1).

“If you do the consultation via telephone, it is hard to actually see how somebody experienced the birth, because you don’t see their facial expressions, you don’t see somebody is trying to hold back tears or something like that” (G8).

#### Impact on future practice

Some midwives were generally negative about the changes that had been introduced due to the COVID-19 pandemic (M5, M7, M9). Others were more positive and found the changes that the pandemic brought to be a good addition to their practices, like for example telemedicine (M1-M6).

Most respondents have also commented on the increase collaboration with colleagues as another great asset to encourage more rapid changes to care pathways.

“If we had a problem, the gynaecologist would be there to back us up. And if they have a problem, we would be there for them … so I hope that that will stay.” (M2)

Furthermore, another midwife wanted to maintain some visitor restrictions during ANC consultations to improve patient-midwife communication, as they found consults were more efficient without small children present (M9).

The gynaecologist’s interviews highlighted that the pandemic led to the introduction of more digital technologies, like video and phone counselling (G1–G9). These changes were mostly seen in a positive way by the participants. The importance of face-to-face appointments was also mentioned. Most preferred the appointment in person instead of video or phone calling. Additionally, home monitoring was improved.

“Definitely, I think doing more online and doing more out of the hospital is the future even in obstetrics care. I don't think it's going to be everything because […] of the condition were actually taken care of, but I think some of it can be definitely transferred.” (G6).

Furthermore, the pandemic led to a more thorough consideration of, and deliberation about, the necessity of certain appointments during the antenatal period, along with a debate over which appointments could be left out of the schedule in the future (G9).

In general, the pandemic made the care process more effective according to the participants.

Moreover, the triage via phone before the appointment facilitated a better preparation and decreased unnecessary visits to the hospital (G9). Additionally, the meetings of the medical team were moved online and were perceived as more informative and effective. Many participants also mentioned the prioritization of ANC as an important lesson learned (G1, G9).

## Discussion

This study investigated the impact of COVID-19 on ANC services in the Netherlands. While recent studies have conducted surveys, interviews were conducted in the present study to explore the topic in-depth [24, 36, 37].

With respect to how antenatal care services have been re-configured at primary care/community level, three significant themes were observed. First, improved hygiene precautions such as wearing facemasks, not shaking hands, and physical distance were prioritized and widely executed. Second, digital technologies such as video appointments and home monitoring were increased in use. These face-to-face appointment alternatives were mostly used for counselling sessions and postpartum visits. Third, the revision to the ANC schedule was highlighted. It was mentioned that care was rationalized to the minimum necessary, but sufficient care would still be provided to pregnant women.

Concerning how antenatal care providers modified their clinical practice during the COVID-19 pandemic, it was found that multiple changes came into effect regarding ANC from both midwives and gynaecologists. Video-calling and telephone consultations were implemented in place of some face-to-face consultations. These ANC variations are consistent with what has been observed in other countries. Jardine et al. [38] reported that 70% of ANC units decreased the number of face-to-face consultations as part of a nationwide survey in the UK examining ANC adjustments, with 89% of practices employing new remote consultation techniques. Another study in the same country found comparable service changes: 51.8% of participants reported routine ANC check-ups shifting to video or phone conversations [39]. Meanwhile, a meta-analysis of seven studies found a 38.6% decrease in face-to-face ANC consultations globally [40]. The increased use of digital technologies was for the most part viewed positively, although the need for face-to-face visits was also emphasized, due to the negative change in the relationship between caregiver and patient as a result of the former. The pandemic sparked a debate over whether all appointments in ANC schedules are necessary to ensure appropriate treatment, as well as over the need for those personal contact visits to develop a relationship between patients and practitioners. In their investigation, Caparros-Gonzalez and Alderdice [22] found that social distancing measures had a detrimental influence on patient’s mental health. Alike, other studies showed a rise in mental health conditions of patients, such as sadness and anxiety [41, 42]. Some studies, on the other hand, implied that telemedicine increases early diagnosis of mental health disorders in pregnant women and is recommended during a pandemic [43]. Nonetheless, according to Galle et al., the introduction of new technologies may exacerbate healthcare access disparities [44]. The research also addressed the immediate problems of telemedicine, such as linguistic limitations, lack of nonverbal feedback and bonding, and patient distrust, all of which are already heightened during the pandemic, as seen in this study too.

Another change that providers have noted and highlighted is the decrease in the overall number of visits to ensure safe care: fewer ultrasounds were typically performed compared to before the pandemic, with greater thought given to whether scans were medically essential. These findings were similar to another Dutch study, which found that pregnant women were more concerned with their safety than with the need for ultrasound scans [24]. Similar trends were observed elsewhere: during the pandemic, the number of ultrasonography visits recorded in both Israel and the US decreased [40]. When comparing NVOG and KNOV guidelines, it is evident that midwife practices had to adjust and modify their guidelines further than hospitals. This might be explained by the organization, experience, and resources of the various care providers. Hospitals may be more prepared for a health crisis than small individual midwife practices, particularly in terms of personnel and financial resources. Furthermore, interviewees put the emphasis on case-by-case determinations, stating that there is no "one-size-fits-all" timetable in ANC because of the nature of the care delivered. As a result, the interviewees stated that there was always the chance that the existing standards may be adjusted to fit each individual scenario.

Visitor restriction, infection control and the use of PPE were mentioned by midwives and gynaecologists in their interviews. Participants saw the restriction of women’s partners and other visitors as a challenge they had with their customers. The measures led to more confrontations with the partners of pregnant women. In some cases, the partners were getting angry about the guideline and could not understand the reasoning behind the rules. A Dutch study found similar results, identifying the visitation limitations as the most significant drawback of ANC modifications by 72.9% of community midwives. Such restrictions happened in the UK as well, with 92% of women being denied the ability to bring visitors to some sessions [39]. Furthermore, it was shown that these restrictions caused great anguish among pregnant women, particularly while visiting emergency consultations. Further research is needed to determine the exact infection risk that visitors pose to midwives, as well as whether such limitations are essential in the long run. So far, there is relatively limited Dutch evidence that visiting care facilities results in increased COVID-19 transmission if local protocols and PPE precautions are rigorously followed [45].

PPE was often cited by respondents in this current study as difficult to get at first, although this improved as the pandemic proceeded. Respondents felt unsupported, and many had to purchase supplies on their own through non-traditional means, with several respondents reporting a lack of national coordination, especially in the first wave. This contrasts with the findings of a national survey in the Netherlands, in which midwives stated that PPE was provided centrally [24]. However, because the van Manen et al. study was a survey, it may have been difficult to fully capture providers’ experiences on the topic.

Some interviewees also stated that pregnant women were delaying seeking care because they were afraid of contracting COVID-19 care providers and had a higher amount of stress and fear during pregnancy, caused by the uncertainty of the pandemic. Other studies have identified similar delays in health-seeking behaviour, including one from the UK, which indicated that 11% of women skipped ANC check-up during the pandemic, frequently due to fear of contracting COVID-19 [39]. Goyal et al. [17] discovered that in India, 32.5% of women had fewer prenatal consultations than recommended during the pandemic, with 33.4% citing fear of contracting COVID-19 as a cause. These shifts in health-seeking behaviour appear to be uneven among countries. Women in Denmark cancelled just 3% of their midwife ANC appointments during the pandemic and preferred to visit midwives in person, possibly suggesting that COVID-19-related worries were lower in this nation [46]. According to López-Morales and colleagues [47], in Argentina, pregnant women had greater rates of sadness and anxiety during the pandemic than not pregnant women. Furthermore, Grumi et al. [48] discussed how this trend may be explained by pandemic-related mental stress and decreased social support as a result of social distancing restrictions.

The scope of this study was limited in terms of recruitment. In fact, participants were recruited based on their desire to engage, which may have resulted in recruitment bias. As a result, the opinions gained may not be reflective of the whole midwife and gynaecologist population. Another barrier to recruitment was the fact that interviews were conducted in English, which resulted in further selection bias based on English competence. The scope of this work only includes the practitioner's viewpoint rather than first-hand observations of the patient. In the future, it is critical to add patients’ perspectives as well. Additionally, participants were interviewed by video call due to COVID-19 constraints. This made research harder to establish a connection with participants, who may have felt less comfortable revealing their experiences as a result.

The diversity of respondents who volunteered to participate in the study was a key strength of this study. Participants were recruited from different areas of the Netherlands, from a mix of rural and urban practices and hospitals, and with varying levels of previous experience. Thus, this study recorded ANC service modifications and attitudes from a varied set of providers spread across the country.

## Conclusions

This qualitative study explored antenatal care providers’ experiences with the changes in the organisation of maternity care and guidelines changes as a result of the COVID-19 pandemic. This event had a massive influence on health-care systems globally. This study reveals that ANC services in the Netherlands changed significantly during COVID-19. Given the risk of future health emergencies, it is critical to learn from the present COVID-19 pandemic and adapt ANC systems, as well as health care systems as a whole, to be better prepared and more resilient.

Evidence from this study suggest that ANC should not be cut back in exchange for other health-care services, and additional capacity should be produced for the whole health system so that the next health crisis does not result in as many shortages; community midwives and gynaecologists should be prioritized for PPE and immunizations. The COVID-19 crisis has also stressed the importance and opportunities of telemedicine and the emphasis on more individualised care.

## Supplementary Information

**Additional file 1.** Document Analysis Data Collection Tool (.doc). Data collection and interpretation tool that was used to carry out the content and thematic analysis of guidelines.

**Additional file 2.** Interview guide for semi-structured interviews with healthcare professionals (midwives, gynaecologists). Interview guide that was developed with and reviewed by ANC experts, and was used to intervie3w participants.

**Additional file 3.** Participant Information Sheet. Information sheet about the current project that was sent prior to the interview to the participants

**Additional file 4.** Informed Consent Form. Informed consent form that was used in the project.

## Data Availability

All data generated or analysed during this study are included in this published article and its supplementary information files.

## Abbreviations

ANC: Antenatal care
COVID-19: Coronavirus Disease 2019
LMIC: Low-middle income country
HIC: High-income country
PPE: Personal protective equipment
NVOG: Nederlandse Vereniging voor Obstetrie en Gynaecologie
KNOV: (Koninklijke Nederlandse Organisatie van Verloskundigen
RIVM: Rijksinstituut voor Volksgezondheiden Milieu
NHG: Nederlands Huisarten Genootschap
