# Supplementary figures and images for: Effect of COVID-19 on antenatal care: experiences of medical professionals in the Netherlands
Source: Reprod Health. 2023 Mar 8;20:40. doi: 10.1186/s12978-023-01587-y (PMC9994402; doi:10.1186/s12978-023-01587-y)

**Additional file 1: Document Analysis Data Collection Tool**


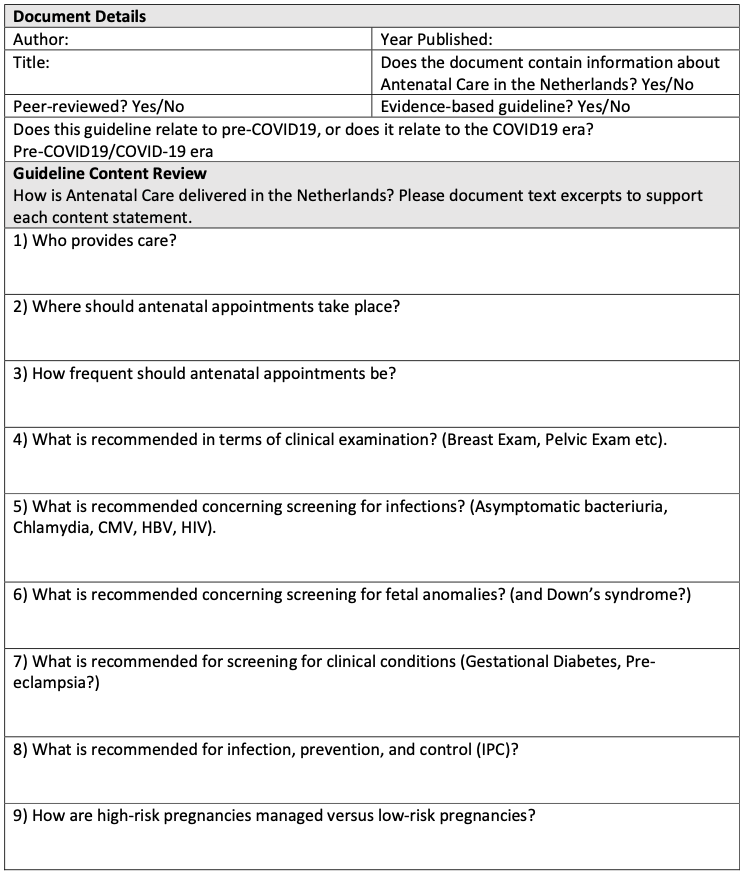

Supplement: Supplementary file 1 — Additional file 1. Document Analysis Data Collection Tool (.doc). Data collection and interpretation tool that was used to carry out the content and thematic analysis of guidelines. [file 12978_2023_1587_MOESM1_ESM.docx]
