# Supplementary material for: Effect of COVID-19 on antenatal care: experiences of medical professionals in the Netherlands
Source: Reprod Health. 2023 Mar 8;20:40. doi: 10.1186/s12978-023-01587-y (PMC9994402; doi:10.1186/s12978-023-01587-y)
Supplement: Supplementary file 2 — Additional file 2. Interview guide for semi-structured interviews with healthcare professionals (midwives, gynaecologists). Interview guide that was developed with and reviewed by ANC experts, and was used to intervie3w participants. [file 12978_2023_1587_MOESM2_ESM.docx]

**Additional file 2: Interview guide for semi-structured interviews with healthcare professionals (midwives, gynaecologists)**

Demographics:

- Name
- Age
- Gender
- Educational background
- Professional experience
- Occupation
- Healthcare facility working and city
- Tenure
- Type of contract (fte)

1. What is your protocol for delivering maternal care in ordinary circumstances (pre-COVID-19)?
2. How does the practice change depend on patients’ characteristics? Please provide examples (e.g., relatively healthy pregnancy vs high-risk situations)
3. Did the pandemic induce a change in the maternal care delivery protocols, at national, regional, or organisational level?
4. How did you adapt your practice/procedures/patient interactions because of the pandemic and in light of the new protocols? Were these protocols fully applicable in the case of your practice or not?
5. Which challenges did you face in adapting your practice of maternal care provision (e.g., introduction and/or enhanced use of digital technologies, adapted working schedules, limited availability of medical equipment or facilities)
6. How did your patients react to such adaptations? Did you observe any challenges or benefits for your patients in adapting to such changes? Did you observe some patients adapting better than others (e.g., based on vulnerability, literacy and/or income level of your patients)?
7. Can you tell me about which changes you observed in the health-seeking behaviour of your patients due to the pandemic (e.g., willingness to attend check-ups)?
8. Did you observe unusual changes in the trend of intakes, or characteristics of your patients, or pregnancy-related risk factors during the pandemic? Were these changes more or less observable among vulnerable groups?
9. Did you observe unusual changes in the general health conditions or health outcomes of your patients since the pandemic started? Were these changes more or less observable among vulnerable groups?
10. Which of the above changes related to intake and health outcomes could be ascribed to pandemic-induced circumstances (e.g., delay of consultations because of lower availability of care provision, lockdown measures)? How did such circumstances disproportionately affect vulnerable groups?
11. Which COVID-induced changes in policy and practice do you think could inform future maternal care provision, to achieve more resilient and equitable care services? What are the “lessons learned”?
