# Supplementary material for: Effect of COVID-19 on antenatal care: experiences of medical professionals in the Netherlands
Source: Reprod Health. 2023 Mar 8;20:40. doi: 10.1186/s12978-023-01587-y (PMC9994402; doi:10.1186/s12978-023-01587-y)
Supplement: Supplementary file 3 — Additional file 3. Participant Information Sheet. Information sheet about the current project that was sent prior to the interview to the participants [file 12978_2023_1587_MOESM3_ESM.docx]

**Additional file 3: Participant Information Sheet**

**Project Title:** The effects of the COVID-19 pandemic on ANC provision (ANC) from a midwife perspective

# Background

On behalf of Maastricht University, Dr. Elena Ambrosino (Supervisor) and Dr. Gareth Evans (MSc student) would like to invite you to take part in the following research project. Before agreeing to take part, please read this information sheet carefully and let us know if anything is unclear or if further information is required.

# What is the purpose of the study?

We are carrying out a research study that investigates the impact of COVID-19 on the provision of community ANC (ANC) in the Netherlands.

As you are aware, COVID-19 has greatly impacted the delivery of all health care services globally, as care providers had to cope with an increase in demand for emergency COVID-19 care, while concurrently needing to maintain safe delivery of essential outpatient services. ANC is one area of care that has been impacted, despite the fact attending antenatal check-ups is essential for pregnant women. Yet, little is known about how exactly ANC provision has changed in the Netherlands, or how the changes have impacted midwives providing those services. This study will therefore focus on examining the effects of COVID-19 on existing care protocols, as well as the impacts on midwife practices.

This study has two main objectives:

**Project Objective 1**: To understand how ANC services have been re-configured at primary care/community level.

**Project Objective 2:** To understand how ANC providers have modified their clinical practice during the COVID-19 pandemic.

# What is involved in participating in this study?

You would be required to take part in a virtual interview that would take no longer than 45 minutes, scheduled at a time most convenient for you. You will be asked questions about how COVID-19 has impacted your ability to provide ANC services to your patients.

# Why have I been invited to take part?

You have been invited to take part, as you are an ANC provider that has worked during the COVID-19 pandemic. You are eligible to participate provided you are a midwife who provides ANC in the community setting in the Netherlands. You must also have worked during the COVID-19 pandemic.

# Do I have to take part?

No, participation is optional. If you choose to take part in this study, you will be given a copy of this information sheet and will be asked to complete a consent form. If you change your mind at any time, you can withdraw your consent to participate without having to provide any reason.

# On what basis will you process my data?

Under the General Data Protection Regulation (GDPR), the University must identify a legal basis for processing personal data and, where appropriate, an additional condition for processing special category data. In line with our charter which states that we advance learning and knowledge by teaching and research, the University processes personal data for research purposes under Article 6 (1) (e) of the GDPR: *Processing is necessary for the performance of a task carried out in the public interest.* Special category data is processed under Article 9 (2) (j): *Processing is necessary for archiving purposes in the public interest, or scientific and historical research purposes or statistical purposes.*

Research will only be undertaken where ethical approval has been obtained, where there is a clear public interest and where appropriate safeguards have been put in place to protect data.

In line with ethical expectations and to comply with common law duty of confidentiality, we will seek your consent to participate where appropriate. This consent will not, however, be our legal basis for processing your data under the GDPR.

# How will you use my data?

This interview will be recorded and transcribed. The results will be published in academic publications or presentations. Statements that might be used in such publications will be anonymized/pseudonymized, i.e., neither your name or the name of your organization will be mentioned or described in a way that the identity can be revealed.

# Will you share my data with 3^rd^ parties?

Data will be accessible to the project team only. For transcription purposes, audio files will be sent to trusted third parties, under the agreement that audio files as well as written transcripts will be immediately deleted once the transcription is completed. Anonymised and/or pseudonymized data may be reused by the research team or other third parties for secondary research purposes.

# How will you keep my data secure?

The University will put in place appropriate technical and organisational measures to protect your personal data and/or special category data. For the purposes of this project, we will store the collected data in a secure academic server. Data transfer across locations will occur only digitally, and only through the secure server. Information will be treated confidentially and shared on a need-to-know basis only. The University is committed to the principle of data protection by design and default and will collect the minimum amount of data necessary for the project. In addition, we will anonymise or pseudonymise personal data.

# Will you transfer my data internationally?

Possibly. Data will safely be stored in password-protected university computers and in university storage solutions.

# Will I be identified in any research outputs?

No. Even when quotes from your interview might be used, these will be presented with pseudonyms concealing the respondents’ identity.

# How long will you keep my data?

Data will be retained in line with legal requirements or where there is a business need. Retention timeframes will be determined in line with the University’s Records Retention Schedule.

# What rights do I have in relation to my data?

Under the GDPR, you have a general right of access to your data, a right to rectification, erasure, restriction, objection, or portability. You also have a right to withdrawal. Please note, not all rights apply where data is processed purely for research purposes.

# Questions or concerns

If you have any questions about this participant information sheet or concerns about how your data is being processed, please contact Dr. Elena Ambrosino (e.ambrosino@maastrichtuniversity.nl) or Carlotta Gamberini (g.gamberini@maastrichtuniversity.nl) in the first instance. For general questions you can contact [privacy@maastrichtuniversity.nl](mailto:privacy@maastrichtuniversity.nl) or [avg-fhml@maastrichtuniversity.nl](mailto:avg-fhml@maastrichtuniversity.nl)
