# Supplementary material for: Effect of COVID-19 on antenatal care: experiences of medical professionals in the Netherlands
Source: Reprod Health. 2023 Mar 8;20:40. doi: 10.1186/s12978-023-01587-y (PMC9994402; doi:10.1186/s12978-023-01587-y)
Supplement: Supplementary file 4 — Additional file 4. Informed Consent Form. Informed consent form that was used in the project. [file 12978_2023_1587_MOESM4_ESM.docx]

**Additional file 4: Informed Consent Form**

**Project Title:** The effects of the COVID-19 pandemic on ANC provision (ANC) from a midwife perspective

**Research Team Contact:**

Dr. Elena Ambrosino e.ambrosino@maastrichtuniversity.nl

Carlotta Gamberini [c.gamberini@maastrichtuniversity.nl](mailto:c.gamberini@maastrichtuniversity.nl)

| This form allows you to state whether you agree to take part in the study. Please read and answer every question. If there is anything you do not understand, or if you want more information, please ask the researcher. |  |
| --- | --- |
| Have you read and understood the information leaflet about the study? | Yes  No |
| Have you had an opportunity to ask questions about the study? | Yes  No |
| Do you understand that the information you provide will be held in confidence by the research team? | Yes  No |
| Do you understand that you may withdraw from the study for any reason, without affecting any services you receive? | Yes  No |
| Do you understand that the information you provide may be used in future research? | Yes  No |
| Do you agree with the use of anonymised/pseudonymised quotes in publications? | Yes  No |
| Do you agree to take part in the study? | Yes  No |
| If yes, do you agree to your interviews being recorded? | Yes  No |

Your name (in BLOCK letters): Click or tap here to enter text.

Your signature: ________________________________________________________________

Interviewer’s name: Carlotta Gamberini
